# Commentary: The Human Default Consciousness and Its Disruption: Insights From an EEG Study of Buddhist Jhāna Meditation

**DOI:** 10.3389/fnhum.2019.00407

**Published:** 2019-11-15

**Authors:** Juergen Fell, Randi von Wrede, Roy Cox

**Affiliations:** Department of Epileptology, University of Bonn, Bonn, Germany

**Keywords:** neural correlate, sleep spindles, slow wave (SW), infraslow oscillation, epileptiform activities, alpha oscillations, artifact

Knowledge of the neural underpinnings of meditation is still fragmentary and inconclusive. There are numerous different kinds of meditation practices and the categorization into major types is difficult. Mixing findings from different meditation practices may dilute outcomes. Moreover, in individuals without meditation experience or in meditation novices it is not clear whether they enter states of consciousness characteristic of meditation during the recording. These issues can be overcome by precisely defining the type of meditation practice under investigation, studying experienced practitioners, and correlating neural outcome measures with total amount of meditation experience.

In a recent issue of Frontiers in Human Neuroscience, Dennison (2019) analyzed the occurrence of different electrophysiological graphoelements (or waveforms) during a type of Buddhist meditation in a large group of experienced practitioners. Besides clearly defining the meditation practice and recruiting experienced practitioners, the author has to be commended for his innovative approach of identifying (and showing) graphoelements, rather than just calculating spectral electroencephalography (EEG) characteristics. As major findings, Dennison (2019) reports that during meditation he detected (1) spindle activity similar to sleep spindles typically observed during sleep stage 2, and (2) infraslow wave activity reminiscent of slow waves characteristic of deep sleep. He interprets these findings as corresponding to the withdrawal from everyday waking consciousness during this type of meditation. Moreover, he describes (3) the occurrence of epileptiform activity in several subjects. These findings seem very exciting at first sight. Unfortunately, they become more dubious after closer scrutiny.

As a first phenomenon, Dennison reports extensive spindling during meditation. Although the author is careful to label these waveforms “meditation spindles” rather than sleep spindles, he nonetheless interprets them to reflect the thalamocortical dynamics characteristic of sleep spindles. However, the presented data strongly suggest that these meditation spindles reflect alpha oscillations. Indeed, alpha activity is typical for waking rest, and like sleep spindles, shows waxing and waning activity patterns. In particular, the spectral power peaks reported by Dennison overlap perfectly with the conventional alpha (8–12 Hz) range (Figure 2, left), with spatial activity profiles typical of posterior alpha sources (Figures 1, 3). In contrast, classical fast sleep spindles are faster (12.5–16 Hz) and have a centro-parietal topography (Zeitlhofer et al., 1997; Cox et al., 2017). Moreover, while the reported frequency range does overlap with the slow sleep spindle range (9–12.5 Hz), slow sleep spindles have a decidedly anterior topography (Zeitlhofer et al., 1997; Cox et al., 2017). Although the author acknowledges some of these differences, he still relates his observations to sleep-like activity. Of note, increased alpha activity is a very common finding across many different meditation practices (for reviews, see e.g., Cahn and Polich, 2006; Fell et al., 2010; Lee et al., 2018), but, as mentioned, alpha is already strongly present during task-free conditions (especially with closed eyes), and may therefore reflect quiet rest and not necessarily meditation-specific processes.

The second phenomenon detected during meditation which the author relates to sleep is what he labels infraslow waves. In contrast to the 0.5–3.5 Hz slow waves typical for deep sleep (comprising both 1 Hz slow oscillations and faster delta components), the oscillations reported by Dennison during meditation have a much lower frequency of around 0.125 Hz. However, this value critically depends on high-pass filter settings and may actually be lower. Again, the author acknowledges this difference, but nonetheless relates this phenomenon to sleep-like activity. Putting that aside, the infraslow wave examples of Figures 4, 5, 7 are, in our opinion, highly suggestive of so-called sweat artifacts (Tatum et al., 2011). Observable in a sizable minority of EEG recordings, these slow, high-amplitude artifacts typically affect a large proportion of channels. Moreover, they do not necessarily affect all channels in a synchronous fashion, thus giving the appearance of “traveling waves.” Alternatively, the author may have been considering so-called infraslow oscillations (typically 0.01–0.2 Hz; for a review, see e.g., Watson, 2018). Importantly, these oscillations occur across both waking and sleep states, and have been shown to modulate higher-frequency activity and cognitive performance (e.g., Vanhatalo et al., 2004; Monto et al., 2008). Thus, we suggest that both Dennison's spindles and infraslow waves reflect established phenomena, which are not specifically linked to sleep.

Regarding the third observation of possible epileptiform activity, the author's Figure 10 shows putative spontaneous spike-wave activity. However, the morphology and clock-like rhythmicity of the depicted patterns is not indicative of epileptiform spike-waves. As a general definition, spike-waves are EEG patterns encompassing a spike followed by a slow wave of ~200–500 ms duration (Weir, 1965; Noachtar et al., 1999). In contrast, the patterns observed by Dennison mostly exhibit bursts of multiple spikes with intermittent fast activity, but no pronounced slow waves. Hence, under the premise that artifacts have been carefully excluded, Figure 10 presents some atypical EEG activity, but in our opinion no clear evidence for epileptiform activity is shown. In contrast, the examples in Figure 11 related to the bodily energization practice are highly suggestive of physiological (in particular, muscle) or technical artifacts commonly encountered with routine EEG recordings (Tatum et al., 2011), and therefore do not allow inferences regarding neural activity.

In summary, the innovative approach and the various phenomena reported by Dennison (2019) are very interesting, but his classification and subsequent interpretation are likely incorrect. As a side note, the described meditation techniques involve control of breathing and it is well-known that pronounced EEG changes occur with altered breathing frequency (e.g., Busek and Kemlink, 2005). More fundamentally, the study fails to unambiguously connect the studied graphoelements—whatever their nature—to meditation. In particular, given that resting state EEG was recorded prior to meditation, it would have been possible to demonstrate differential electrophysiological activity between these two brain states. Absent this comparison, or comparison with an adequate control group, it is not possible to answer whether the studied waveforms relate specifically to the state of meditation, or the state of quiet wakefulness more broadly.

## Author Contributions

All authors listed have made a substantial, direct and intellectual contribution to the work, and approved it for publication.

### Conflict of Interest

The authors declare that the research was conducted in the absence of any commercial or financial relationships that could be construed as a potential conflict of interest.
